# Quality of Life in Patients with Dementia with Lewy Bodies

**DOI:** 10.1155/2018/8320901

**Published:** 2018-07-05

**Authors:** Chuo-Yu Lee, Shih-Jung Cheng, Hui-Chi Lin, Yu-Lu Liao, Pei-Hao Chen

**Affiliations:** ^1^Department of Neurology, Mackay Memorial Hospital, Taipei, Taiwan; ^2^Graduate Institute of Chemistry, Tamkang University, New Taipei City, Taiwan; ^3^Department of Medicine, Mackay Medical College, New Taipei City, Taiwan; ^4^Department of Physical Therapy and Assistive Technology, National Yang-Ming University, Taipei, Taiwan; ^5^Department of Accounting Information, Takming University of Science and Technology, Taipei, Taiwan; ^6^Graduate Institute of Mechanical and Electrical Engineering, National Taipei University of Technology, Taipei, Taiwan

## Abstract

Dementia with Lewy bodies (DLB) is a complex, multisymptom disorder. When making decisions regarding the treatment of DLB, the patient's quality of life (QoL) should always be the main consideration. To our knowledge, this is the first review article focusing on the QoL in DLB patients. We searched the PubMed database using the keywords “quality of life” and “dementia with Lewy bodies.” Previously, no specific instrument had been developed for assessing the QoL in DLB patients. Patients with DLB have a decreased QoL compared to patients with Alzheimer's disease, which is reportedly caused by several factors including level of independence in instrumental activities of daily living, whether the patient is living with the caregiver, apathy, delusion, and dysautonomia. The direct effect of visual hallucination, sleep, and movement disorders on the QoL in DLB patients has not been previously studied. The role of cognitive function on the QoL is still controversial. In a randomized controlled study, memantine may improve the QoL in PDD or DLB patients. We concluded that it is important to develop a specific instrument to assess the QoL in DLB patients. Furthermore, there is an urgent need for large clinical trials to identify factors associated with the QoL and how they can be managed.

## 1. Introduction

Dementia with Lewy bodies (DLB) is the second most common neurodegenerative dementia after Alzheimer's disease (AD) in people over 65 years of age. A recent published population-based cohort study from the Taiwan National Health Insurance Research Database showed the incidence of DLB was 7.10 per 100,000 person-years [1]. DLB differs from AD as early cognitive symptoms include deficits in visuospatial and executive function, rather than memory. DLB is characterized by parkinsonism, fluctuations in mental status, visual hallucination, and hypersensitivity to neuroleptics. Therefore, it is difficult to discriminate between DLB and Parkinson's disease with dementia (PDD). Both diseases also share a similar pathologic finding, Lewy bodies. Generally, if cognitive impairments appear within a year of parkinsonism, DLB is diagnosed, while patients with parkinsonism for at least a year prior to cognitive impairment are classified as PDD. Early amyloid deposition in DLB relative to PDD may explain the difference in the timing of dementia and parkinsonism [2].

The quality of life (QoL) is a key outcome measure of health and social service interventions. Currently, patient-reported outcome measures are increasingly used in evaluating health and social care. Definitions of health-related QoL include physical, mental, social, and role functioning and health perceptions [3]. Because aging is a global issue, it is critical to identify QoL determinants in the elderly who suffer from chronic disease. A recent study from Southern Taiwan showed Alzheimer disease-8, a screening tool, had the strongest association with the total QoL score in 115 old-age adults suffering from chronic disease. It is important for geriatric health care providers to realize that cognitive impairment among old age adults with chronic disease is a critical determining factor of poor QoL [4].

Assessing the QoL in people with dementia is crucial for evaluating their outcomes; however, it is challenging to interview patients who have a limited ability to express themselves and may lack insight. Meanwhile, there are questions concerning the validity of generic measures of QoL, which are not specific to dementia. Currently, evaluations of QoL largely focus on general dementia or AD. A systematic review [5] regarding the QoL in dementia strongly suggests that depression is consistently related to a decreased QoL. Surprisingly, there is no convincing evidence indicating that lower cognitive ability or greater limitations in the activity are associated with a lower QoL.

Evidence concerning the QoL in DLB patients is limited [6]. In a comparison study, the QoL was measured using the *Alzheimer Disease-Related Quality of Life* (ADRQL) evaluation, a proxy-rated, dementia-specific instrument. The authors concluded that DLB patients have a poorer QoL than AD patients [7]. Figari et al. compared the QoL in 46 AD, 23 DLB, and 39 Huntington's disease (HD) patients, all of whom had dementia for at least two years. Patients with DLB scored significantly lower on the SF-12 Physical and Mental Health Summary than patients with HD and AD. The authors concluded that, when adjusted for age, cognition, comorbidity, and depression, patients with DLB had the poorest QoL [8].

There is only one study whose primary aim was to compare the QoL in patients with DLB and AD [9]. 34 DLB patients and 34 cognitive-matched AD patients were evaluated using two QoL instruments: EQ-5D and QoL-AD. The results showed both patient-rated QoL and proxy-rated QoL were poorer in patients with DLB than those with AD. Due to many differences in clinical symptoms, it is reasonable to predict differences in the QOL between diseases. Herein, we elaborate on studies related to the QoL in DLB patients.

## 2. Methods

Studies were selected from the PubMed database using keywords “quality of life” and “dementia with Lewy bodies.” All languages (with an English abstract) were included. Articles spanned the period between June 1986 and December 2017. Searches yielded 102 citations of which 21 were included as relevant. Other data were collected through references from selected articles and searching relevant journals offline.

### 2.1. Dementia-Specific QoL Measurement Scales

Unfortunately, there are no instruments available to specifically evaluate the QoL in DLB patients. Therefore, we herein discuss QOL measurement scales for dementia.

There is some debate regarding whether to use the patient's or caregiver's reported QoL as the measurement. Typically, the QoL is rated higher by patients than by caregiver proxy measures. The differences are associated with increased levels of caregiver burden and caregiver depression, rather than lower levels of patients' cognitive performance [10]. Therefore, it is reasonable to directly assess subjective QoL in patients with mild to moderate dementia. Interestingly, Karlawish et al. proposed a method to minimize the discrepancy between patient's and caregiver's QoL scores: caregivers should rate using substituted judgment as if they were the patients [11]. Issues regarding the exclusion of patients with severe dementia from self-reported questionnaires and weak reliability of proxy versions remain unresolved.

There is limited knowledge regarding the standard instruments used to evaluate the QoL in dementia patients. In a recently published review [12], the most frequently used dementia-specific instrument was the Quality of Life in Alzheimer's Disease (QOL-AD) and Dementia Quality of Life questionnaire (DEMQOL). As for generic measures, EuroQol 5-dimension (EQ-5D) and Short Form surveys (SF-36 or SF-12) were widely used. The authors recommended dementia-specific DEMQOL, generic SF-12, and health utility EQ-5D-5L, based on both self- and proxy-report [12]. In the following paragraphs, we will briefly introduce each widely-used measurement scale.

### 2.2. Quality of Life in Alzheimer's Disease (QOL-AD)

The QOL-AD [13] is an instrument specifically developed for mild to severe dementia. It includes 13 items, including physical health, energy, mood, living situation, memory, family, marriage, friends, self, ability to do chores, ability to do things for fun, money, and life as a whole. Each domain is rated from 1 (poor) to 4 (excellent). As for the caregiver version, which contains 15 items, marriage and money are removed, while people who work here, ability to take care of oneself, ability to live with others, and ability to make choices in one's life are added.

### 2.3. Dementia Quality of Life Questionnaire (DEMQOL)

The DEMQOL [14] aims to assess QoL in mild to moderate dementia. It includes 5 domains including daily activities and looking after self, health and well-being, cognitive functioning, social relationships, self-concept, and a total of 28 items. Each item is scored from 1 to 4. A proxy version has been developed for caregivers, DEMQOL-Proxy, which contains 31 items.

### 2.4. EuroQol 5-Dimension (EQ-5D-5L)

The EQ-5D-5L [15] is a generic instrument. Respondents are asked to rate their current health status on 5 dimensions: mobility, hygiene, usual activities, pain/discomfort, and anxiety/depression. For each dimension, the respondent gives 1 of 5 possible ratings according to symptom severity. The questionnaire also includes a visual analog scale (VAS) as a secondary part, ranging from 0 (death) to 100 (perfect health). Thus, two QoL values are acquired with this instrument.

### 2.5. Short Form-36 and Short Form-12 (SF-36 and SF-12)

The SF-36 [16] is a generic measurement containing 36 questions across 8 dimensions of health status: vitality, physical functioning, bodily pain, general health perceptions, physical role functioning, emotional role functioning, social role functioning, and mental health. A shorter version, SF-12, was developed to minimize respondent burden if only physical and mental health summary scores are of interest.

A summary of the four widely-used scales is provided in Table 1.

### 2.6. Reported Symptoms That May Affect QoL in DLB Patients

One study compared the QoL in patients with DLB and AD as its primary aim and demonstrated that the following are important determinants of the QoL in DLB patients: their Neuropsychiatric Inventory score and level of independency in instrumental activities of daily living (ADL), whether the patient lives with the caregiver, apathy, and delusion [9]. Similar to most findings in overall dementia, cognitive function has no strong relation to the QoL in this study.

Caregivers play an important role in affecting a patient's QoL. In a cross-sectional study [17] of 161 patients with dementia (including 13 DLB patients), the QoL was measured using the ADRQL. The results indicated that some predictors of a reduced QoL include behavioral and depressive symptoms, dependency in basic ADL, poorer cognitive function, use of antipsychotic medication, caregiver burden, and caregiver not being an adult child. The role of cognitive function on the QoL is still controversial. Here, we will discuss some reported causative factors for a poorer QoL in DLB patients.

### 2.7. Visual Hallucinations

Visual hallucinations occur in 60 to 70% of DLB patients, usually beginning in the first 2 or 3 years of the disease [18]. The presentation of visual hallucinations in the first 4 years following the onset of dementia has a positive and negative predictive value for DLB of 81% and 79%, respectively [19]. The most common visual hallucinations are fully formed persons (84%), animals or bugs (37%), and objects (39%) [20]. Early misperceptions and misidentification of family members are usually reported in DLB. Capgras syndrome, a delusion where a person has been substituted by an imposter with a similar outward, is also common in DLB and is associated with higher rates of visual hallucinations and anxiety [21]. A recently published postmortem study [22] showed DLB cases had reduced neuronal density in the intermediate gray layer of the superior colliculus tissue, a structure important for directing attention toward visual targets. This finding may provide pathologic evidence for visual hallucinations in DLB.

A cross-sectional and retrospective study of 1025 patients with dementia in Spain found that delusion and hallucinations were more prevalent in DLB patients than those with AD and PDD [23]. In a recent comparative study of 207 delusional and nondelusional patients with DLB, the authors concluded that the delusional patients had poorer cognitive function and more severe neuropsychiatric symptoms [24]. In a cross-sectional study, 21 DLB and 35 PDD patients with recurrent visual hallucinations were evaluated. Most patients had complex hallucinations daily, usually lasting minutes. The study showed that neuropsychiatric symptoms that coexist with hallucinations are apathy, sleep disturbance, and anxiety [25]. The direct effect of visual hallucination on the QoL in DLB patients has not been previously studied.

Treatment of psychosis is challenging in DLB, since many patients are hypersensitive to neuroleptic drugs; therefore, nonpharmacologic treatment approaches should be considered first. Typical antipsychotic agents should be avoided, while the evidence for atypical antipsychotics is controversial. At present, quetiapine and clozapine are usually prescribed. Pimavanserin, a selective serotonin 5-HT2A inverse agonist, has beneficial effects on treating psychosis in PDD [26], while similar results are expected in DLB patients.

### 2.8. Depression and Apathy

The presence of depression and other behavioral and psychological symptoms of dementia (BPSD) may worsen the QoL of dementia patients and their caregivers [27]. The BPSD can be classified into four clusters: depression, aggressive behaviors, psychosis, and euphoria. In AD and DLB patients, a cross-sectional analysis determined that depressive symptom in patients may be the most important BPSD symptom as it was the only one shown to cause depression in caregivers [28].

A previous literature revealed that DLB is associated with higher scores on the Geriatric Depression Scale compared to AD, and a higher rate of depression is found in the early stages of the disorder [29]. Kurisu et al. compared the QoL of 279 degenerative dementia patients with different subtypes by using the *QOL Questionnaire for Dementia* (QOL-D) as an evaluation scale. The QOL-D comprises six domains: positive affect, negative affect and actions, communication, restlessness, attachment to others, and spontaneity. Results showed that apathy in frontotemporal dementia (FTD) and DLB patients and depression in DLB patients might cause the reduced positive affect in FTD and DLB patients compared to AD patients [30]. Apathy also plays an important role in evaluating the QoL in DLB patients. In a DLB and AD comparison study, DLB patients are less able to complete the QoL questionnaire [6].

In DLB, the presence of Lewy bodies in limbic, paralimbic, and neocortical regions may account for the appearance of depressive symptoms [27]. Early detection of depression in DLB patients is important as these symptoms are treatable. In a recently published review, the authors concluded that neuropsychiatric symptoms, especially psychosis and depression, are priority targets for intervention to improve the outcome of patients with DLB [6].

### 2.9. Sleep Disorders

Sleep disorders are common in DLB patients, with both rapid eye movement sleep behavior disorder (RBD) and fluctuating cognition being part of the clinical diagnostic criteria. In a contemporary review in sleep medicine, the authors concluded that appropriate management of sleep-related symptoms can improve the QoL in patients with neurodegenerative disorders [31]. Orexins (also known as hypocretins) are secreted in neurons in the lateral hypothalamus that are related to sleep regulation and attention [32]. Lower levels of orexin-1 were detected in the cerebrospinal fluid of DLB patients compared to AD patients and control subjects [33].

76% of DLB patients had RBD [34], which is characterized by acting out dreams, resulting in vocalizations and even violent behavior. Other nocturnal symptoms such as anxiety, periodic leg movements, urinary dysfunction, and difficulty turning over in bed can contribute to sleep problems [31]. In a retrospective study of 78 patients with DLB and sleep disorders who underwent polysomnography, approximately three quarters experienced many arousals not accounted for by a movement or breathing disturbance. Among patients who did not show evidence of significantly disordered breathing, 62% of arousals were arousals for no apparent reason [35]. The direct effect of sleep disorders on the QoL in DLB patients has not been previously studied.

### 2.10. Dysautonomia

In a study [36] of the prevalence of autonomic symptoms in dementia, the authors concluded that total autonomic symptom scores [37], urinary symptoms, constipation, and postural dizziness were significantly higher in DLB patients than in AD patients. By using the SF-36 [38] as a measurement, higher autonomic symptom scores were related to a lower QoL. The authors proposed that the effect of autonomic symptoms upon the QoL may be due to the limitations in ADL.

A systematic review of autonomic dysfunction in *α*-synucleinopathies revealed that cardiovascular autonomic failure has a significant impact on daily activities and QoL. Cerebral white matter changes in image and cognitive decline may be related to altered cerebral perfusion, vascular pressure stress, and associated disruption of the blood-brain barrier [39]. In *α*-synucleinopathies, autonomic dysfunction is more severe in DLB than in PD patients [40].

Both preganglionic and postganglionic dysfunctions may be present in DLB patients [41]. In a retrospective examination of DLB patients, urinary incontinence and constipation were the most commonly presented autonomic symptoms, occurring in 97 and 83% of patients, respectively, while syncope occurred in 28% of patients [42]. Orthostatic hypotension (OH) has been reported in around 50% of DLB patients [43]. OH is defined as the reduction in systolic blood pressure of at least 20 mmHg or diastolic blood pressure of 10 mmHg within 3 minutes of standing from the lying position [44]. Patients may not present with classic postural dizziness, but instead nonspecific malaise or lethargy, which can markedly increase the risk of falls or syncope. Management may include salt supplementation, compression stockings, or oral medication such as midodrine or fludrocortisone [45]. Furthermore, a higher prevalence of carotid sinus syndrome, an altered arterial sinus response to baroceptive stimulation resulting in syncope, was found in 32% of DLB patients compared to 11.1% of AD patients [46].

### 2.11. Reported Management That May Improve QoL in DLB Patients

It can be helpful to divide the symptoms of DLB into five categories: cognitive, neuropsychiatric, movement, autonomic, and sleep. It is important to take a detailed history and form a comprehensive treatment strategy to improve a patient's QoL [47]. There is little published data regarding the effect of the management of symptoms on the QoL in DLB patients.

### 2.12. Armodafinil

Because armodafinil has a longer half-life than modafinil, we may predict its better effect at treating patients with excessive daytime sleepiness [48]. In a 12-week pilot trial of oral armodafinil therapy (120–250 mg daily), DLB patients with hypersomnia showed improved scores on the Epworth Sleepiness Scale, Maintenance of Wakefulness Test, and Clinical Global Impression of Change. Moreover, caregivers' overall QoL improved at week 12 [49]. It should be noted that the effect of armodafinil on patients' QoL was not analyzed in this trial.

### 2.13. Memantine

Memantine, an NMDA receptor antagonist, has been widely used in treating moderate to severe AD. A randomized controlled study was conducted on 70 patients with PDD or DLB over 24 weeks using caregiver-rated QOL-AD as a measurement. A secondary analysis of this study showed that memantine improved the total QoL, body function, physical health, energy, mood, and memory when compared to the placebo group [50]. Large-scale trials are required to confirm these preliminary findings. On the other hand, although cholinesterase inhibitors are widely prescribed for patients with DLB, we found no reported effect of cholinesterase inhibitors on the QoL in DLB patients.

### 2.14. Exercise

Exercise may improve functional outcomes in PD and AD patients. The multisymptom nature of the DLB (parkinsonism, cognitive, and psychiatric) results in these patients often being excluded from clinical trials to avoid confounding results. A recent systematic review confirms a scarcity of research investigating exercise as a potential therapy in DLB patients [51]. The authors concluded that the effect of exercise on cognitive, psychiatric, QoL, and physiological outcomes remains unclear in DLB patients. Further clinical trials in larger cohorts are necessary.

## 3. Conclusions

This is, to our knowledge, the first review article focusing on the QoL in patients with dementia with Lewy bodies. Our review may not be fully comprehensive due to the limited literature available regarding this topic. Physicians should keep the patient's QoL in the forefront when managing their symptoms. The following are reported factors leading to poorer QoL in DLB patients: independence in instrumental ADL, whether the patient is living with the caregiver, the presence of depression, apathy, delusion, and dysautonomia.

First, we concluded that it is important to develop a specific instrument to assess the QoL in DLB patients. Apathy is more common in DLB patients, causing them less able to complete the questionnaire [6]. Therefore, it is reasonable to design a scale with proxy version. Since DLB is a complex, multisymptom disorder, we recommend the following domains should be taken into account: health and well-being, daily activities, cognitive functioning, positive and negative affect, restless behavior, social interaction, and satisfaction (i.e., restful sleep). Second, we hope that with the advent of more diagnostic criteria and advances in biomarkers, there will be well-defined DLB cohorts for clinical trials. Further studies may be facilitated by international or multicenter cooperation. For example, a large longitudinal cohort of 5624 DLB patients has been developed recently in 135 dementia centers in Italy (the DLB-SINdem study group) [52]. There is an urgent need for large, cross-sectional, and longitudinal trials to determine other factors associated with the QoL, such as cognition, visual hallucination, sleep, and movement disorders. Finally, because of the poor prognosis and high socioeconomic burden of DBL, we should consider the health-related QoL as one of the outcome measurements when developing new drugs for DLB.

## Figures and Tables

**Table 1:** Characteristics of QoL measurement scales for dementia.

Instrument	Patient report	Proxy report	Domains	Items	Measurement	Brief summary
Quality of Life in Alzheimer's Disease (QOL-AD)	Yes	Yes	Physical health Mental health Social and functional domains	13 (15 for proxy version)	Four-point scale score Range 13–52 for patient report and range 15–60 for proxy report Higher scores equal to higher QoL	The best researched of all the dementia measurement scales Relatively brief Suitable for mild to severe dementia
Dementia Quality of Life questionnaire (DEMQOL)	Yes	Yes	Daily activities and looking after self Health and well-being Cognitive functioning Social relationships Self-concept	28 (31 for proxy version)	Four-point scale score Range 28–112 for patient report and range 31–124 for proxy report Higher scores equal to higher QoL	The conceptual framework is based on health-related QoL, with less social relevance The proxy version performed well in psychometric tests Suitable for mild to moderate dementia
EuroQol 5-dimension (EQ-5D-5L)	Yes	No	1st part: Mobility Hygiene Usual activities Pain/discomfort Anxiety/depression 2nd part: Visual analogue scale (VAS)	No	1st part: having no problems for 1, having slight problems for 2, having moderate problems for 3, having severe problems for 4, and being unable to do/having extreme problems for 5 2nd part: 0 for worst; 100 for best health status	A generic, preference-based instrument for health related QoL The 5-digit number for 5 domains can be converted into a single weighted index score Appropriate in people with mild to moderate dementia
Short Form-36 (SF-36)	Yes	No	Vitality Physical functioning Bodily pain General health perceptions Physical role functioning Emotional role functioning Social role functioning Mental health	36	One score in each domain, calculated by special software, representing weighted sums of the questions (0 equivalent to maximum disability; 100 equivalent to no disability)	A generic health status measure A shorter version, SF-12, was developed to minimize respondent burden if only physical and mental health summary scores are of interest
